# Confined Mobility of TonB and FepA in *Escherichia coli* Membranes

**DOI:** 10.1371/journal.pone.0160862

**Published:** 2016-12-09

**Authors:** Yoriko Lill, Lorne D. Jordan, Chuck R. Smallwood, Salete M. Newton, Markus A. Lill, Phillip E. Klebba, Ken Ritchie

**Affiliations:** 1 Department of Physics, Purdue University, West Lafayette, Indiana, United States of America; 2 Department of Biochemistry and Molecular Biophysics, Kansas State University, Manhattan, Kansas, United States of America; 3 Department of Chemistry and Biochemistry, University of Oklahoma, Norman, Oklahoma, United States of America; 4 Department of Medicinal Chemistry and Molecular Pharmacology, Purdue University, West Lafayette, Indiana, United States of America; Centre National de la Recherche Scientifique, Aix-Marseille Université, FRANCE

## Abstract

The important process of nutrient uptake in *Escherichia coli*, in many cases, involves transit of the nutrient through a class of beta-barrel proteins in the outer membrane known as TonB-dependent transporters (TBDTs) and requires interaction with the inner membrane protein TonB. Here we have imaged the mobility of the ferric enterobactin transporter FepA and TonB by tracking them in the membranes of live *E*. *coli* with single-molecule resolution at time-scales ranging from milliseconds to seconds. We employed simple simulations to model/analyze the lateral diffusion in the membranes of *E*.*coli*, to take into account both the highly curved geometry of the cell and artifactual effects expected due to finite exposure time imaging. We find that both molecules perform confined lateral diffusion in their respective membranes in the absence of ligand with FepA confined to a region $0.180_{-0.007}^{+0.006}$ μm in radius in the outer membrane and TonB confined to a region $0.266_{-0.009}^{+0.007}$ μm in radius in the inner membrane. The diffusion coefficient of these molecules on millisecond time-scales was estimated to be $21_{-5}^{+9}$ μm^2^/s and $5.4_{-0.8}^{+1.5}$ μm^2^/s for FepA and TonB, respectively, implying that each molecule is free to diffuse within its domain. Disruption of the inner membrane potential, deletion of ExbB/D from the inner membrane, presence of ligand or antibody to FepA and disruption of the MreB cytoskeleton was all found to further restrict the mobility of both molecules. Results are analyzed in terms of changes in confinement size and interactions between the two proteins.

## Introduction

The important process of nutrient uptake in *Escherichia coli* requires transport across the lipopolysaccharide (LPS)-rich outer membrane (OM), passage through the periplasmic space that contains the peptidoglycan (PG), and finally transport across the cell’s inner membrane (IM), that surrounds the cytoplasm. In many cases, the first step in this process involves transit through a class of beta-barrel proteins in the OM known as TonB-dependent transporters (TBDTs). Certain uptake pathways require interactions between TBDT and the IM protein TonB, via an undetermined, energy-dependent mechanism [1, 2]. Structural studies showed direct interactions between the C-terminus of TonB [3, 4] and a domain in TBDT called the TonB box.

TBDTs are energy-dependent gated channels that usually transport large metal complexes which cannot fit through porins, and are too scarce to enter by mass-action-driven transport. The number of TBDTs varies among different bacteria, from 7 demonstrated TBDTs in *E*. *coli* to 65 predicted TBDTs in *Caulobacter crescentus* [5]. In *E*. *coli* TBDTs scavenge and bind micronutrients with high affinities, especially iron chelates (called siderophores), but also vitamin B12, and they are parasitized by colicins, phages, and naturally occurring antibiotics [5]. In other bacteria they were reported to serve as receptors for nickel complexes and even carbohydrates [6]. Siderophores are microbial iron chelators that themselves bind iron with high affinity to solubilize Fe^3+^ in the environment. The cognate receptors for ferric enterobactin, ferrichrome and ferric citrate, FepA, FhuA and FecA respectively, are examples of *E*. *coli* TBDT. A typical TBDT spans the OM as an amphipathic 22-stranded β-barrel of about 50 Å in diameter with long extracellular loops, and a globular N-terminal domain that fills the barrel [7]. The 150-residue N terminus blocks uptake through the barrel; it is held by hydrogen bonds and polar contacts with the interior wall of the barrel [8] and it must move or rearrange for transport to occur. Binding of specific substrates on the extracellular surface of TBDT relocates the TonB box region on the periplasmic surface of the TBDT, allowing its interaction with the C-terminus of TonB [5]. Ferric enterobactin transport through FepA may involve dislodgement of the plug domain from the B-barrel (ball-and-chain model), or formation of a smaller diffusion channel (transient pore model) [9, 10]. Site-directed alkylation experiments indicated that the N-terminal domain of FepA at least in part dislodges into the periplasm to allow passage of ferric enterobactin [10].

**Fig 1:**
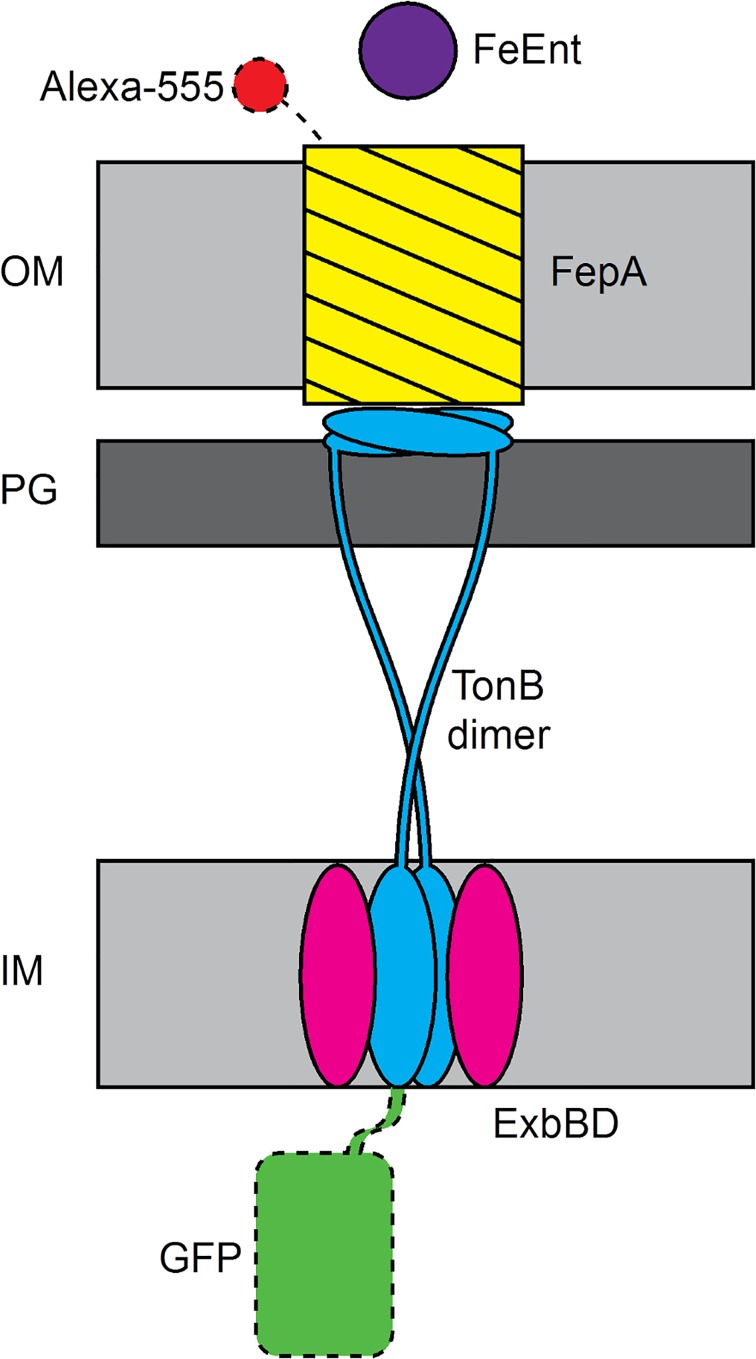
Schematic of the interaction between the TonB-ExbB-ExbD complex in the inner membrane and its interaction with the Ferric enterobactin (FeEnt) TBDT FepA in the outer membrane of *E*. *coli*. In this study, either FepA was labeled extracellularly with Alexa-555 or TonB was labeled intracellularly with GFP. IM: inner membrane, PG: peptidoglycan layer, OM: outer membrane.

Energy-dependent uptake through TBDTs requires interaction with TonB in complex with ExbB and ExbD in the IM (see Fig 1 for a schematic representation of this system). TonB contains a proline-rich stretch of about 100 amino acids that may interact with ExbD, a globular C-terminus that binds the TonB-box of TBDT, and a hydrophobic, transmembrane N-terminus domain that may interact with ExbB [11]. Crystal structures described the C-terminus of TonB in dimeric form [12, 13], and in monomeric form in complex with OM transporters [3, 4]. The monomeric TonB C-terminus was also characterized by NMR in solution [14]. The TonB-ExbB-ExbD complex is thought to derive energy from proton-motive force across the IM, and transmit it to the OM [15]. ExbD was predicted to have a similar structure to TonB, with its N-terminal domain spanning the IM and the majority of its sequence residing in the periplasm [16]. In higher abundance, ExbB was predicted to mainly reside on the cytoplasmic side of the IM, with three transmembrane domains. The exact stoichiometry of the TonB-ExbBD oligomer is unknown, but recent evidence suggests an ExbB_4_ExbD_2_ complex [17]. Mechanistically, it was shown that TonB remains in the inner membrane and does not shuttle across the periplasmic space during activity as was once proposed [18, 19]. Thus, the N-terminal transmembrane domain of TonB remains resident in the IM, but downstream regions of the TonB polypeptide cross the periplasmic space to interact with TBDTs. ExbBD show sequence homology to the flagellar “stator” proteins MotAB [20], which inferred a rotational motion by TonB that was supported by measurements of GFP-TonB anisotropy [21]. Hence, TonB may pull or twist the N-termini of TBDTs to promote transport of substrates into the periplasm. ATP-binding-cassette (ABC) transporters subsequently move the metal complexes through the periplasm and IM.

The technique of single-molecule and single particle tracking has been used for the past 15 years to probe structure and interactions on length scales ranging from tens of nanometers to micrometers in the membranes of bacterial cells. The earliest study of outer membrane protein mobility using single particle tracking was performed by Oddershede et al. [22], observing the diffusion of LamB in live *E*. *coli*. Since then several studies have employed fluorescent probes to observe the molecular mobility in bacteria at the single molecule level. Specifically, high-speed single-molecule tracking (SMT) has been employed to observe GFP or Kaede labeled protein diffusion in the *E*. *coli* cytoplasm at observation rates up to 250 Hz [23, 24] and up to 1000 Hz [25] and in the inner membrane of *E*. *coli* at rates up to 1000 Hz [26]. Leake et al. has employed single molecule methods to monitor the mobility and stoichiometry of TatA in the twin-arginine translocation (Tat) system and of MotB of the bacterial cytoplasmic membrane with fluorescent proteins [27, 28]. Rassam et al. tracked colicin labeled BtuB and Cir in the outer membrane of E. coli and found both proteins confined in domains approx. 0.5 μm diameter domains [29]. A recent report has also studied TcpP in V. cholerae cells using a super-resolution imaging with photoactivated localization microscopy (PALM) and single-molecule tracking to reveal localization and mobility [30].

Here, we conducted single molecule imaging to measure the mobility of the ferric enterobactin transporter FepA and TonB in the membranes of live *E*. *coli* at time-scales ranging from milliseconds to seconds. In an iron deficient environment, as many as 35,000 copies/cell of chromosomally encoded FepA exist in the OM [19], as opposed to TonB, which occurs at a maximum concentration of 1000 copies/cell [31]. Thus, the low rate of ferric siderophore uptake (about 5 per min; [19, 32, 33]), together with the finding that all FepA proteins in the OM transport ferric enterobactin [34], suggest that the rate limiting factor in the uptake is TonB’s capacity to find and facilitate ligand-bound OM transporters. We discovered that both proteins show confined diffusion in their respective membranes. At high time resolutions, the diffusion coefficient for each protein was similar, implying essentially free motion within their confining regions. The movement of FepA occurred in a much smaller region than that of TonB. We also investigated the effect of membrane potential on the mobility of FepA and TonB, as well as the effect of ExbB/ExbD deletion on TonB mobility. Our results show small effects on the lateral mobility of FepA and TonB through disruption of the inner membrane potential. Deletion of ExbB/ExbD only slightly altered the diffusion behavior of TonB as well. The presence of ferric enterobactin in solution slowed the lateral mobility of FepA and TonB. We conducted simple simulations to model/analyze the lateral diffusion in the membranes of *E*.*coli*, to take into account both the highly curved geometry of the cell and artifactual effects expected due to finite exposure time imaging [35].

## Materials and Methods

### Bacterial strains

*E*.*coli* strain BN1071 (F-, entA, pro, trp, B1) harbored plasmid pGT, that encodes GFP-TonB [19]. In this construct, a mutant version of *Aequorea vitoria* GFP (sg-GFP, pQbioT7-GFP from QbioGene, Irvine, CA, which contains substitutions F64L, S65C, I167T) was cloned in frame between the natural *tonB* promoter and the wild-type *tonB* structural on the low copy vector pHSG575 [19, 36], to produce pGT. GFP-TonB has normal TonB activity, including ferric siderophore uptake and colicin susceptibility [19]. OKN23, which lacks *exbB* and *exbD*, was derived also from BN1071 by site-directed deletion mutagenesis. The doubling time of BN1071, the parent *E*. *coli* strain, is ~60 min in iron deficient minimal media [37]. Both the growth of its derivative strain OKN3/pGT, and its rate of ^59^FeEnt transport, are indistinguishable from that of BN1071 [19].

### Fluorescence labeling of FepAS271C/FepAA698C

OKN3 (*ΔfepA*) is a derivative of *E*.*coli* K-12 strain BN1071 that we transformed with plasmids harboring wild-type *fepA* or its mutant derivatives. We used site-directed Cys substitution mutations to create *fepA* alleles *S271C* and *A698C* on pITS23, a derivative of pHSG675 that carries wild-type *fepA* under its natural promoter [10, 38, 39]. We modified the resulting cys residues with Alexa 488 or 546 maleimide in live cells. FepA is the only OM protein that is significantly labeled by these procedures [10, 38]. The strain OKN3/pGT/pITS23*fepAS271C* also grows and transports FeEnt just like BN1071 [21, 38].

## Antibody against FepA

Anti-FepA monoclonal antibodies (MAb 44, MAb 45) were diluted from ascitic fluids and used at 1/200 [38, 40]. They recognize FepA surface epitopes within residues 290–339, and block binding of both ferric enterobactin and colicin B. We labeled them with Alexa-555 using the reagents and protocol of Invitrogen/Molecular Probes (Eugene, OR).

### Ferric Enterobactin

FeEnt was prepared and purified as previously described [38, 41].

### Growth of cells

After overnight growth at *E*. *coli* strains in Luria-Bertani (LB) media supplemented with streptomycin and chloramphenicol, we subcultured them at 1% into morpholinopropanesulfonate (MOPS)-buffered minimal medium [42] without iron, to incubate a further 5–7 hours at 37°C. For experiments with A22 (S-(3,4- Dichlorobenzyl)isothiourea), the MOPS medium contained A22 at 30μg/ml during this incubation. The cells labeled with Alexa 488/546 were kept on ice for 1–2 days until they were revived at room temperature in MOPS medium for >1 hour before single-molecule imaging.

### Sample preparation for single-molecule imaging

Glass-bottom cover dishes were used as sample chambers. The cover dishes were immersed in 5% Contrad detergent overnight. After 30 min of sonication, they were immersed in 0.1 M KOH overnight, sonicated 30 min, and rinsed in clean water. Prior to use, 100 μl of 0.1 mg/ml poly-L-lysine (M.W 70,000–150,000, Sigma) was allowed to adsorb to the dried cover glass. Excess poly-L-lysine solution was removed and the cover glass was rinsed with clean water. *E*. *coli* cells were plated in the chamber and allowed to adhere to the poly-L-lysine layer for 20 min in MOPS minimal medium. Excess nonadherent cells were washed away with phosphate buffered saline (PBS).

### Single-molecule measurement

Imaging was performed using oblique angle laser illuminated epifluorescence microscopy. An argon-ion (488 nm, Spectra Physics, Newport, Irvine, CA) or He-Ne (543 nm, Research Electro-Optics, Inc., Boulder, CO) laser was directed by a dichroic mirror (Chroma Technology, Bellows Falls, VT) off the optical axis through the objective (1.45 NA oil immersion, Olympus America, Melville, NY) to illuminate only the adherent cells instead of the entire sample chamber. Fluorescence emission was collected through the dichroic mirror and an emission filter (500–550 nm or 562.5–637.5-nm, Chroma) on a dual multichannel-plate intensified, Peltier-cooled CCD camera (Turbo-120Z, Stanford Photonics, Palo Alto, CA) acquired as a continuous stream of 30 – 1000Hz without delay between images. Image magnification was set to have the pixel resolution to be about 50 nm/pixel at the camera. The sample with *E*.*coli* cells in PBS were kept at approximately 32°C during the measurements which lasted up to 1 hour. Presence of doubling cells showed their viability under these condition and there was no evidence of changes in the measured properties throughout the observation time. For the experiments with the proton ionophore CCCP, cells were observed in PBS containing 0.5 mM CCCP. To observe the effect of antibody or ligand binding to FepA on its own or TonB diffusion, we added monoclonal antibody against FepA to 0.1 mg/ml or FeEnt to about 1 μM, respectively, in PBS.

Data collection was performed as follows. Cells in a field of view were illuminated initially for a few minutes to photobleach existing fluorescence in the cell to aid in imaging single molecules. Trajectories were then collected by illuminating the cell for only a few seconds more. Laser powers ranged from 30–0.3 W/cm^2^. To avoid effects of this laser illumination, we observed each field of view once, then moved to a new area of the dish. Only those fluorescence signals which showed single step photobleaching and or blinking during the observation time were used in the analysis. The illumination power was set at each observation frame rate to keep the signal-to-noise ratio, defined as $(I_{s}-I_{b})/\sqrt{{\sigma^{2}}_{s}+{\sigma^{2}}_{b}}$, where *I*_*s*_ and *I*_*b*_ are the sums and *σ*^2^_*s*_ and *σ*^2^_*b*_ are the variances in the sum of intensity in a 9×9 pixel box surrounding the signal and neighboring the signal, respectively, to approximately 2 to maximize the lifetime of the fluorescence signal.

### Single-molecule analysis

The video images that contained signal from single GFPs or Alexa 488/546 were analyzed to follow diffusion. The limited focal depth of the single molecule microscopy set up and an illumination off the optical axis through the objective allowed detection of relatively thin section in the plane of the sample. The apparent position of the fluorescent molecules in the video image was determined in two-dimension as described in previous studies [43]. Data in which fluorescent molecules appeared at least ten consecutive frames were employed in the analysis. To analyze molecular movement quantitatively, the mean-square displacement (MSD), ⟨*r*^2^(*t*)⟩, of the observed fluorescent molecules was calculated for each trajectory. All the average MSD at each time delay was determined by averaging over the MSD calculated for each trajectory obtained from each experiment.

Individual fluorescent molecules were imaged diffusing in live *E*. *coli* and tracked to determine their trajectories. The imaging was done at rates of 30, 40, 60, 120, 260, 400 and 1000 Hz and data was collected at each rate for at least 10 frames. For observing longer time range, additional imaging was done at rates 1, 2, 5, 10, and 20 Hz. The MSD of fluorescent molecules, averaged over all trajectories measured at that rate, vs. time delay was determined at each rate. For each frame rate in each of the observed conditions, at least 70 (except for FepA in the presence of CCCP, 20–100) trajectories were used in the averaging. In order to produce a single, combined MSD curve over all rates, the y-intercept of a linear regression through the first six MSD points at 1000 Hz was set to the origin and then each successive MSD curve's y-intercept was set so as to make a continuous MSD curve over all time scales. We determined microscopic diffusion coefficients by assuming simple Brownian motion for the short time range. The MSD should follow a linear dependence on time according to ⟨*r*^2^⟩ = 4*Dt* + *δ*^2^, where *D*, the effective diffusion coefficient can be determined by a linear fit to the average MSD versus time delay. *δ*^2^ is due to the finite accuracy with which the particle position can be measured. From our measured *δ*^2^ we find our precision in localization to be about 50 nm at 1000 Hz. Since the data collected at the rates slower than 60 Hz did not show any change in diffusion behavior at the longer time scale, we have employed only the data obtained at 60 Hz and faster for detailed analysis.

### Monte Carlo simulation

A program described in previous study was modified to perform Monte Carlo simulations of 2-dimensional TonB and FepA diffusion in the membrane [25]. Briefly, at the beginning of each run of the simulation, a single molecule, represented as point particle, was randomly placed on the surface of an ideal *E*. *coli* cell (a cylinder whose length equals its diameter with hemispherical end caps). Since we observed most TonB molecules within the cylindrical portion of *E*.*coli*, we only allowed the simulations for TonB diffusion to reside in the cylindrical portion of the ideal cell, employing reflective boundary conditions at the edges of the cylinder. The diffusion of FepA was modeled on the entire simulated cell surface. Steps were then taken on the surface in random directions with lengths taken from a normal distribution whose width depends on the simulated particle’s diffusion coefficient. Confinement was implemented by centering a circular area of given radius on the surface about the point particle’s initial position with reflective boundary conditions.

Simulations were run internally at a time scale of 0.5 ms per step. To simulate blurring effects due to the finite exposure time of the camera, sequential positions obtained in the simulation were averaged to reproduce measurements taken at the experimental frame rates. For each frame rate and simulation condition (diffusion coefficient and confinement radius), 1000 trajectories were collected. Simulations were analyzed for each frame rate used in the same manner as the experimental measurements and the computed combined MSD was compared to the observed combined MSD. Fitting was performed by minimizing the reduced Chi-squared value between the simulated and observed combined MSD for varying diffusion coefficient and confinement size. The vertical shift for each simulated frame rate was left as a free parameter in the fitting to the observed combined MSD. The reduced chi-squared surface over a range of diffusion coefficients and confinement sizes for all the experimental data are shown as supporting figures (S4 Fig).

## Results

### Spatial Distribution of TonB and FepA

**Fig 2:**
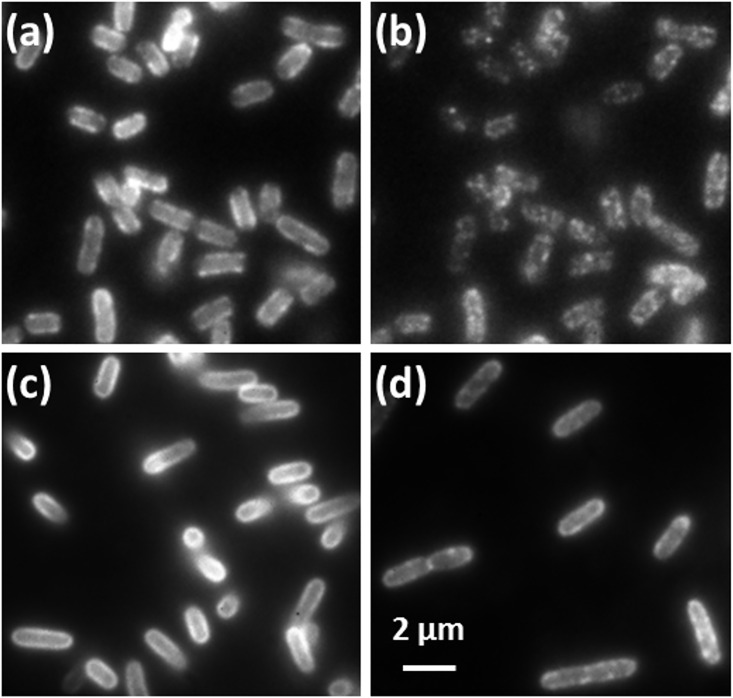
**Images of the distribution of TonB (a, b) and FepA (c, d) in the membranes of *E*. *coli* at high (a, c) and low (b, d) concentrations of active fluorescent markers.** At low concentration, both proteins show apparent clustering in their respective membranes.

Initial Observation of GFP-TonB and Alexa-labeled FepA containing cells, prior to any photobleaching, is shown in Fig 2. Treatment of the cells with the membrane depolarizing drug CCCP or the cytoskeleton disrupting drug A22 did not affect the localization patterns. Further, the cell strain lacking ExbB/D also showed no change in the localization of TonB.

Reduction in overall intensity through photobleaching of this initial, bright fluorescence signal for GFP-TonB and Alexa-labeled FepA containing cells is shown in Fig 2B and 2D. TonB shows apparent clustering as the total intensity of fluorescence is reduced under all conditions we have studied in this paper. In comparison, Alexa-labeled FepA showed a faint (low contrast) indication of clustering during photobleaching of the initial fluorescence. Due to the small expected size of these apparent clusters (see single molecule tracking results below) and the highly curved nature of the cells, quantitative analysis of these images was not performed.

### Mobility of TonB and FepA

**Fig 3:**
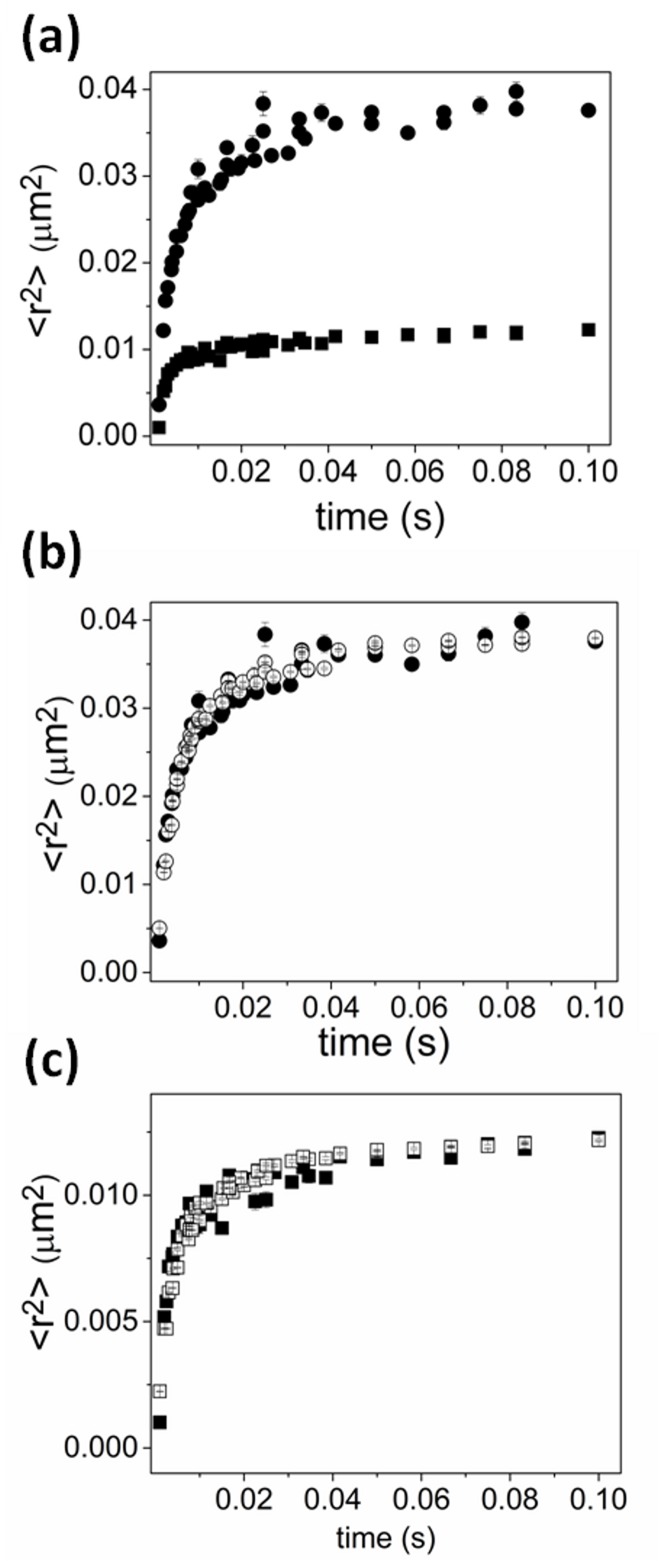
MSD of TonB and of FepA. a) MSD of TonB (solid circles) and of FepA (solid squares). b) Fit to confined diffusion model for TonB (data: solid circles, fit: open circles) which gives best fit parameters of a confinement radius of $0.266_{-0.009}^{+0.007}$ μm with short-time (microsecond timescale) diffusion coefficient in the range $5.4_{-0.8}^{+1.5}$ μm^2^/s. c) Fit to confined diffusion model for FepA (data: solid squares, fit: open squares) which gives best fit parameters of a confinement radius of $0.180_{-0.007}^{+0.006}$ μm with short-time (microsecond timescale) diffusion coefficient in of $21_{-5}^{+9}$ μm^2^/s.

We observed the lateral mobility of GFP-TonB and Alexa-FepA in the absence of FeEnt through direct imaging of individual molecules. The motions of both molecules were confined in regions much smaller than the cell dimension (Fig 3A). From the average maximum mean-square displacement (MSD) of each molecule during observation, which should asymptotically approach ⟨*r*^2^⟩ = *L*^2^/3 in 2-D where *L* is the confinement diameter [44], TonB was confined within a radius of about 0.2 μm, and FepA within about 0.1 μm, respectively. They reached their respective boundaries within about 50 ms and 10 ms, respectively. We did not observe any change in the confined diffusion behavior over 10 s observation time. Note that one can compare directly the mobility here with that of cytoplasmic GFP in the same cellular system in reference (25) to see that we are observing a unique mobility due to GFP-TonB being membrane bound.

The observed average MSDs of TonB and FepA in their membranes were modeled using Monte Carlo simulations of diffusive motion over an idealized 3-D cell surface projected onto a 2-D “camera” as is in the actual experiment. The diffusion of TonB and FepA was well described with models employing confinements that restrict Brownian motion within about $0.266_{-0.009}^{+0.007}$ μm and $0.180_{-0.007}^{+0.006}$ μm in radius, respectively with short-time (microsecond timescale) diffusion coefficients set in the range $5.4_{-0.8}^{+1.5}$ μm^2^/s for TonB and $21_{-5}^{+9}$ μm^2^/s for FepA (Fig 3B and 3C). Neither varying cell sizes nor implementing the presence of obstacles in the membrane improved the fit.

### Mobility of TonB and FepA under various conditions and treatments

In what follows we will model changes in the mobility of FepA by the simulation above, reporting changes in confinement size and microscopic diffusion coefficient. For changes in the mobility of TonB, we will employ two models in the analysis. First we will, as for FepA, use the above simulation method to determine possible changes in confinement size and microscopic diffusion coefficient. The second model, will assume that changes in TonB mobility and confinement may be due to differential interaction with the more highly confined FepA. Changes in confinement is then modeled as a mixture of a fraction of TonB diffusing as TonB does in the wild-type cell in the absence of added FeEnt and a fraction that diffuses as the more highly confined FepA does due to interaction. This is accomplished by using the experimental results of the last section as standard curves (Fig 3) and fitting the observed MSD for TonB under a given condition as a mixture of the two standard curves.

**Fig 4:**
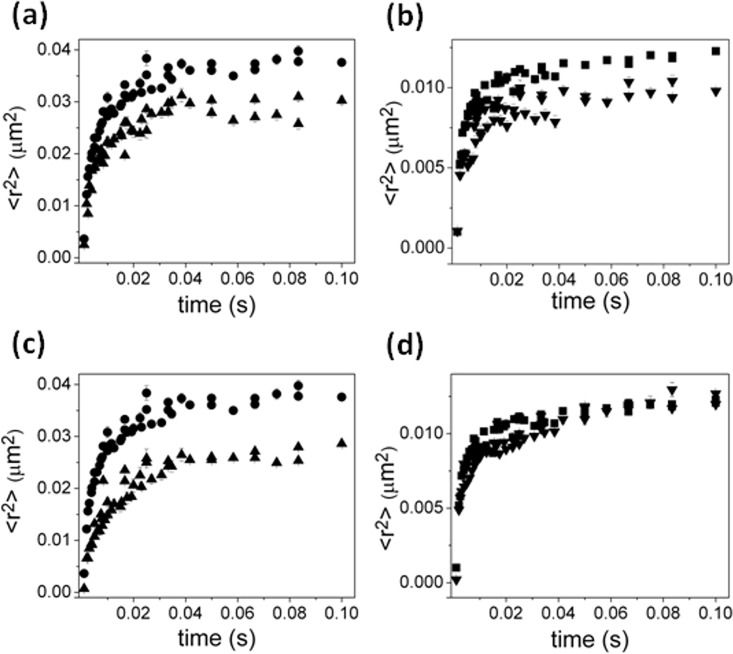
Effect of CCCP and ligand FeEnt on MSDs of TonB and FepA. TonB in the presence (triangles) and absence (circles) of (a) CCCP or (c) FeEnt. FepA in the presence (upside-down triangles) and absence (squares) of (b) CCCP or (d) FeEnt. Fitting suggests that the effect of CCCP and the ligand FeEnt on the mobility of TonB is a reduction in the diffusion coefficient and confinement size and a reduction in confinement size for FepA.

The proton ionophore CCCP dissipates the proton-motive force (PMF) in membranes, and is used to investigate energy-dependent mechanisms in cells. Previous studies showed that CCCP prevents transport of substrates by TBDTs [34, 45, 46], and we looked at the mobility of both TonB (Fig 4A) and FepA (Fig 4B) in CCCP-treated cells. Simulation of TonB diffusion implied a similar confinement size of $0.240_{-0.030}^{+0.010}$ μm radius with a diffusion coefficient of $3.8_{-1.3}^{+2.6}$ μm^2^/s at short times (S1A Fig). The MSD of TonB motion was also fit using a weighted average of the MSD for TonB and FepA in the absence of CCCP (S1B Fig) revealing a best fit when the curve consisted of 65 ± 5% the MSD for TonB and 35 ± 5% the MSD for FepA. The mobility of FepA changed slightly in the presence of CCCP, with a diffusion coefficient of $27_{-6}^{+13}$ μm^2^/s and a confinement radius of $0.150_{-0.014}^{+0.015}$ μm, comparable to the confinement size in untreated cells (S1C Fig).

To check the effect of the siderophore FeEnt on the mobility of both FepA and TonB, their motion was imaged in the presence of an excess, saturating amount of FeEnt. While FeEnt had little apparent effect on FepA mobility in the OM (Fig 4D), its presence and binding to FepA in the OM restricted TonB motion in the IM. Fitting by the confinement model suggests that diffusion coefficient of FepA is $20_{-7}^{+11}$ μm^2^/s with a marginal reduction in confinement size to $0.160_{-0.002}^{+0.002}$ μm, whereas the mobility of TonB was decreased overall to a diffusion coefficient of $3.8_{-0.6}^{+0.7}$ μm^2^/s and a confinement radius of $0.186_{-0.007}^{+0.004}$ μm in the presence of FeEnt as shown in Fig 4C and 4D. If we instead assume a fraction of TonB is bound to FepA and hence moves as if confined in the same domain FepA is confined in, the MSD for TonB can be fitted by a weighted average of the MSD of TonB (53 ± 3%) in the absence of FeEnt and 47 ± 3% the MSD for FepA.

**Fig 5:**
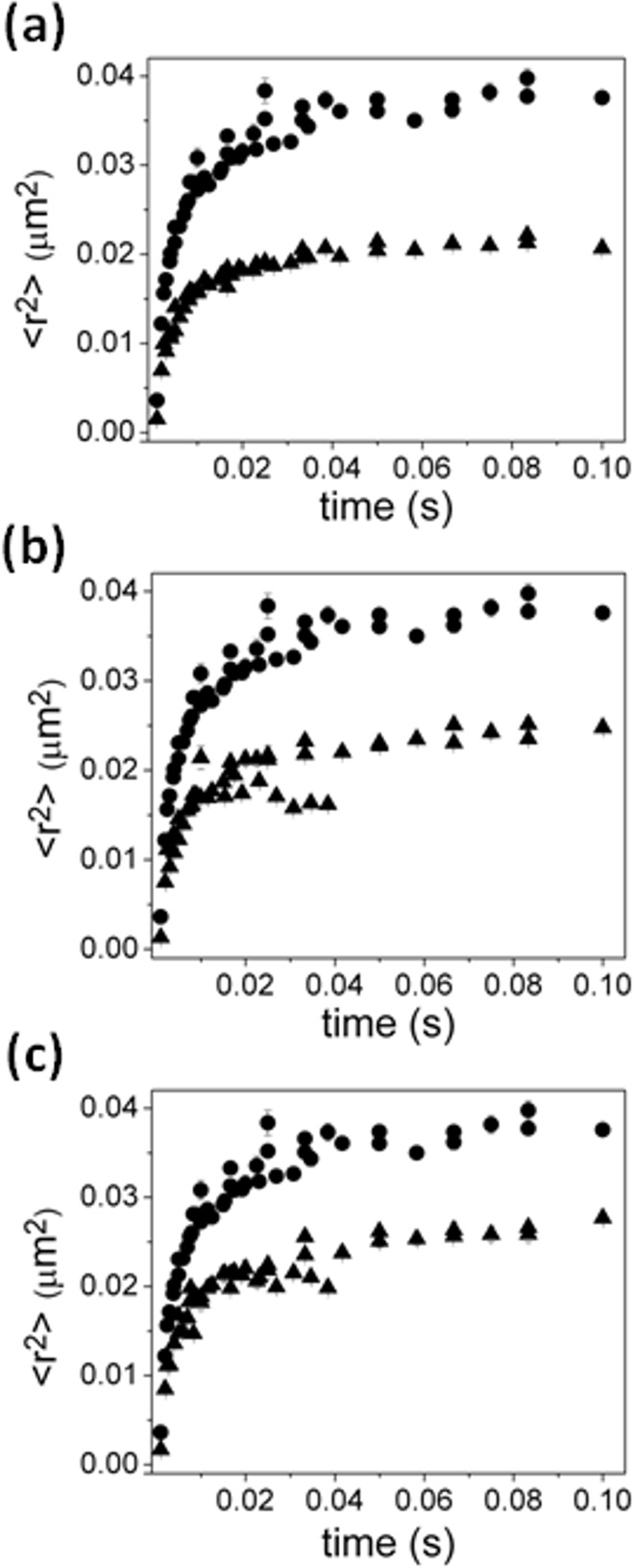
**Effect of (a) deletion of ExbB/D (triangles), (b) presence of monoclonal antibody against FepA (triangles), and (c) presence of MreB–disrupting drug A22 (triangles) on MSDs of TonB (circles).** Fitting suggests that the effect of deletion of ExbB/D and disruption of MreB is to reduce the size of confinement of TonB while the effect of anti-FepA is to increase the mobility of TonB which appears as a decrease in confinement size.

In cells lacking ExbB and ExbD, which participate with TonB in energy acquisition from the electrochemical gradient across the IM, the lateral mobility of TonB was more restricted as well (Fig 5A). Fitting the MSD with a combination of the MSD’s from TonB and FepA in wild-type cells revealed a best fit when the curve consisted of 35 ± 2% the MSD for TonB and 65 ± 2% the MSD for FepAs. The best simulation assuming changes in confinement size and diffusion coefficient to fit to the experimental data from *E*.*coli* without ExbB and ExbD was with a confinement size set to $0.178_{-0.007}^{+0.004}$ μm in radius and a short-time diffusion coefficient at $1.4_{-0.1}^{+0.2}$ μm^2^/s (S3A Fig).

The mobility of GFP-TonB was also observed while OM-resident FepA was exposed to anti-FepA MAbs (Fig 5B). The binding of anti-FepA MAbs 44 or 45 [40] blocks FeEnt uptake and induces conformational changes in FepA. The resulting MSD plot best fit a mixture of 43 ± 3% of the MSD of baseline TonB and 57 ± 3% for the MSD for FepA. Simulations suggested a reduced confinement size ($0.200_{-0.010}^{+0.007}$ μm) compared to the absence of the antibody against FepA, but an increased diffusion coefficient $10.0_{-1.5}^{+1.0}$ μm^2^/s (S3C Fig).

To test if the MreB-based cytoskeleton was responsible for confining TonB, we observed the mobility of TonB in the presence of the MreB-disrupting drug A22 [47, 48]. There was a measured effect in the MSD of TonB in cells treated with A22 compared to that of untreated cells (Fig 5C). In these cells, rounded due to disruption of MreB polymerization, TonB diffusion was modeled with a confinement of $0.183_{-0.006}^{+0.003}$ μm and diffusion coefficient of $6.3_{-0.9}^{+1.6}$ μm^2^/s. The results of all fits are summarized in Tables 1 and 2.

**Table 1:** Parameters to fit to variable confinement size model

Molecule/Condition	Diffusion coefficient (μm^2^/s)	Confinement in radius (μm)
TonB	$5.4_{-0.8}^{+1.5}$	$0.266_{-0.009}^{+0.007}$
TonB/CCCP	$3.8_{-1.3}^{+2.6}$	$0.240_{-0.030}^{+0.010}$
TonB/Δ*exbb/d*	$1.4_{-0.1}^{+0.2}$	$0.178_{-0.007}^{+0.004}$
TonB/anti-FepA	$10.0_{-1.5}^{+1.0}$	$0.200_{-0.010}^{+0.007}$
TonB/A22	$6.3_{-0.9}^{+1.6}$	$0.183_{-0.006}^{+0.003}$
TonB/FeEnt	$3.8_{-0.6}^{+0.7}$	$0.186_{-0.007}^{+0.004}$
FepA	$21_{-5}^{+9}$	$0.180_{-0.007}^{+0.006}$
FepA/CCCP	$27_{-6}^{+13}$	$0.150_{-0.014}^{+0.015}$
FepA/FeEnt	$20_{-7}^{+11}$	$0.160_{-0.002}^{+0.002}$

**Table 2:** Parameters to fit mixed diffusion mode model

Molecule/Condition	% FepA	% TonB
TonB/CCCP	35 ± 5	65 ± 5
TonB/Δ*exbb/d*	65 ± 2	35 ± 2
TonB/anti-FepA	57 ± 3	43 ± 3
TonB/A22	$48_{-4}^{+3}$	$52_{-3}^{+4}$
TonB/FeEnt	47 ± 3	53 ± 3
FepA/CCCP	$100_{-0.10}^{+0}$	$0_{-0}^{+0.10}$
FepA/FeEnt	$100_{-0.33}^{+0}$	$0_{-0}^{+0.33}$

## Discussion

Lateral diffusion of TonB in the inner membrane and FepA in the outer membrane, observed by single-molecule tracking showed confined mobility. Simulation of Brownian motion in the highly curved membrane in confinement domains well described the experimental data that were fitted to the model by employing diffusion coefficients similar in order of magnitude to that from earlier single-molecule studies on *E*.*coli* membrane proteins [22, 49, 50]. From the simulated data, the diffusion coefficient of TonB was estimated as about $5.4_{-0.8}^{+1.5}$ μm^2^/s, with a compartment size of about $0.266_{-0.009}^{+0.007}$ μm in radius. FepA’s diffusion coefficient was found in the range of $21_{-5}^{+9}$ μm^2^/s with a confinement size of $0.180_{-0.007}^{+0.006}$ μm in radius. The confinement of both proteins was consistent with the apparent clustered distribution of these proteins we observed when we viewed higher concentrations of the labeled proteins in the membranes.

Assuming that the above results represent the base diffusion properties of TonB and FepA in their respective membranes under ligand free conditions, we also analyzed the mobility of each protein under varying conditions in light of possible interactions between them. Because the mobility of FepA was essentially unchanged in the conditions we tested, and because FepA was confined to a smaller region than TonB, interactions between the two proteins may be revealed as a shift in the confinement of TonB from its larger domain to the smaller domain of FepA. As such, we analyzed the mobility of TonB under different conditions as a mixture of the mobilities of FepA and TonB under ligand-free conditions. Under ligand-free conditions, TonB may still interact transiently with FepA but since it is confined in a larger region than FepA these interactions must be transient. A shift in confinement of TonB to smaller areas would imply the appearance of longer lasting interactions with FepA.

In this analysis, the effect of membrane depolarization by CCCP was minimal with the MSD of TonB split: 35 ± 5% of TonB molecules behaved like FepA and 65 ± 5% like undisturbed TonB. In cells lacking ExbB and ExbD, TonB’s mobility was split into 65 ± 2% like FepA and 35 ± 2% like undisturbed TonB. These results imply that TonB may interact more strongly with FepA in the absence of ExbB/D, thus restricting the motion of a large portion of TonB to the domain that delimits FepA.

A recent study of the motion of GFP-TonB in the IM, measured by fluorescence anisotropy, showed restriction in GFP-TonB movement compared to freely diffusing cytoplasmic GFP [21]. Disruption of membrane potential (necessary for transport of iron across the OM) decreased the rotational motion of TonB. On the other hand, deletion of the proposed energy harvesting complex ExbB/D that presumably allows TonB to acquire energy for transport from the membrane potential, increased the rotational mobility of TonB. Our data show that in cells treated with CCCP, or lacking ExbBD (*ΔexbBD*), the lateral mobility of TonB was more restricted under both conditions. Upon addition of the cognate ligand FeEnt, while FepA’s mobility was decreased in terms of diffusion coefficient while keeping the confinement size relatively unchanged, TonB’s MSD become more restricted overall. Interpreting this result in terms of interactions, FepA’s MSD was essentially unchanged, whereas TonB’s MSD could be fitted as a mixture between 47 ± 3% of FepA’s MSD and 53 ± 3% of ligand-free TonB’s MSD. The former subpopulation diffusing like ligand-free FepA may be a consequence of the interaction between TonB and FepA during FeEnt transport. The data suggest that about 1/2 of the time TonB was free from, or only transiently interacting with FepA, whereas the other half of the time TonB was bound to FepA. The addition of anti-FepA MAbs that recognized the receptor’s ligand binding site restricted TonB’s motion slightly more, such that 57 ± 3% moved like FepA and only 43 ± 3% moved like baseline TonB. Because these antisera inhibit FeEnt uptake [40], it is likely that they simulate ligand binding, but without subsequent transport, and thereby cause long-lived interactions between TonB and FepA.

These findings taken together imply that interaction between TonB and FepA may be investigated through the mobility of TonB, which changes in response to association with FepA. The timescale at which we observed dynamics allowed us to detect determinants of diffusion behavior along the membrane that are structural in nature, as well as from molecular interactions. A method to observe mobility a at smaller timescale would allow detection of molecular interaction effects in more detail, to further dissect the step-by-step mechanism of ligand transport through the FepA–TonB/ExbBD complex.

Finally, to test if the MreB-based cytoskeleton was responsible for the structure confining TonB, we imaged mobility in cells in the presence of A22. TonB diffusion in these cells had a confinement of $0.183_{-0.006}^{+0.003}$ μm and diffusion coefficient of $6.3_{-0.9}^{+1.6}$ μm^2^/s. The decrease in confinement size of TonB in these A22 treated cells despite the disruption of MreB polymerization suggests that the cytoplasmic structure is most likely not the cause of confined lateral mobility of TonB in the cytoplasmic membrane. The limiting factor leading to relatively immobile TonB maybe the result of mesh like structure of peptidoglycan in the periplasm keeping the C-terminus of TonB in confined regions.

## Supporting Information

S1 Fig Cells treated with membrane potential disrupting CCCP. Fit to the MSD of TonB (data: solid triangles, fit: open triangles) by (a) confinement model and (b) assuming a mixture of the MSD for ligand-free TonB (Fig 3B) and FepA (Fig 3C). Fit to the MSD of FepA (data: solid upside-down triangles, fit: open upside-down triangles) (c) confinement model and (d) assuming a mixture of the MSD for ligand-free TonB (Fig 3B) and FepA (Fig 3C).
(TIF)

S2 Fig TonB and FepA mobility in the presence of FeEnt. Fit to the MSD of TonB (data: solid triangles, fit: open triangles) by (a) confinement model and (b) assuming a mixture of the MSD for ligand-free TonB (Fig 3B) and FepA (Fig 3C). Fit to the MSD of FepA (data: solid upside-down triangles, fit: open upside-down triangles) (c) confinement model and (d) assuming a mixture of the MSD for ligand-free TonB (Fig 3B) and FepA (Fig 3C).
(TIF)

S3 Fig Model fits to the MSD of TonB. Fit to the MSD of TonB (data: solid triangles, fit: open triangles) by (a,c,e) confinement model and (b,d,e) assuming a mixture of the MSD for ligand-free TonB (Fig 3B) and FepA (Fig 3C) for cells (a,b) lacking ExbB/D, (c,d) in the presence of anti-FepA and (e,f) in the presence of MreB disrupting A22.
(TIF)

S4 Fig Reduced chi-squared distribution between the MSD of the models and the experimental data. Reduced chi-squared surfaces from Monte Carlo simulation fits to the observed MSDs: ligand-free (a) TonB and (b) FepA, (c, d) TonB and FepA in the presence of FeEnt, (e, f) TonB and FepA in the cells treated with CCCP, (g) TonB for the cells lacking ExbB/D, and TonB in the presence of (h) anti-FepA antibody and (i) A22. Reduced chi-squared distribution for models assuming a mixture of the MSD for ligand-free TonB (Fig 3B) and FepA (Fig 3C): (j, k) TonB and FepA in the presence of FeEnt, (l, m) TonB and FepA in the cells treated with CCCP, (n) TonB for the cells lacking ExbB/D, and TonB in the presence of (o) anti-FepA antibody and (p) A22.
(PDF)

## References

[1] Ollis AA, Manning M, Held KG, Postle K. Cytoplasmic membrane protonmotive force energizes periplasmic interactions between ExbD and TonB. Mol Microbiol. 2009;73(3):466–81. 10.1111/j.1365-2958.2009.06785.x 19627500 PMC2729267

[2] Postle K, Larsen RA. TonB-dependent energy transduction between outer and cytoplasmic membranes. Biometals. 2007;20(3–4):453–65. 10.1007/s10534-006-9071-6 17225934

[3] Pawelek PD, Croteau N, Ng-Thow-Hing C, Khursigara CM, Moiseeva N, Allaire M, et al Structure of TonB in complex with FhuA, E. coli outer membrane receptor. Science. 2006;312(5778):1399–402. 10.1126/science.1128057 16741125

[4] Shultis DD, Purdy MD, Banchs CN, Wiener MC. Outer membrane active transport: structure of the BtuB:TonB complex. Science. 2006;312(5778):1396–9. 10.1126/science.1127694 16741124

[5] Noinaj N, Guillier M, Barnard TJ, Buchanan SK. TonB-dependent transporters: regulation, structure, and function. Annu Rev Microbiol. 2010;64:43–60. 10.1146/annurev.micro.112408.134247 20420522 PMC3108441

[6] Schauer K, Rodionov DA, de Reuse H. New substrates for TonB-dependent transport: do we only see the 'tip of the iceberg'? Trends Biochem Sci. 2008;33(7):330–8. 10.1016/j.tibs.2008.04.012 18539464

[7] Nikaido H. Molecular basis of bacterial outer membrane permeability revisited. Microbiol Mol Biol Rev. 2003;67(4):593–656. 10.1128/MMBR.67.4.593-656.2003 14665678 PMC309051

[8] Faraldo-Gomez JD, Sansom MS. Acquisition of siderophores in gram-negative bacteria. Nat Rev Mol Cell Biol. 2003;4(2):105–16. 10.1038/nrm1015 12563288

[9] Buchanan SK, Smith BS, Venkatramani L, Xia D, Esser L, Palnitkar M, et al Crystal structure of the outer membrane active transporter FepA from Escherichia coli. Nat Struct Biol. 1999;6(1):56–63. 10.1038/4931 9886293

[10] Ma L, Kaserer W, Annamalai R, Scott DC, Jin B, Jiang X, et al Evidence of ball-and-chain transport of ferric enterobactin through FepA. J Biol Chem. 2007;282(1):397–406. 10.1074/jbc.M605333200 17056600 PMC2398697

[11] Kohler SD, Weber A, Howard SP, Welte W, Drescher M. The proline-rich domain of TonB possesses an extended polyproline II-like conformation of sufficient length to span the periplasm of Gram-negative bacteria. Protein Sci. 2010;19(4):625–30. 10.1002/pro.345 20095050 PMC2867004

[12] Chang C, Mooser A, Pluckthun A, Wlodawer A. Crystal structure of the dimeric C-terminal domain of TonB reveals a novel fold. J Biol Chem. 2001;276(29):27535–40. 10.1074/jbc.M102778200 11328822

[13] Kodding J, Killig F, Polzer P, Howard SP, Diederichs K, Welte W. Crystal structure of a 92-residue C-terminal fragment of TonB from Escherichia coli reveals significant conformational changes compared to structures of smaller TonB fragments. J Biol Chem. 2005;280(4):3022–8. 10.1074/jbc.M411155200 15522863

[14] Peacock RS, Andrushchenko VV, Demcoe AR, Gehmlich M, Lu LS, Herrero AG, et al Characterization of TonB interactions with the FepA cork domain and FecA N-terminal signaling domain. Biometals. 2006;19(2):127–42. 10.1007/s10534-005-5420-0 16718599

[15] Ollis AA, Kumar A, Postle K. The ExbD periplasmic domain contains distinct functional regions for two stages in TonB energization. Journal of bacteriology. 2012;194(12):3069–77. 10.1128/JB.00015-12 22493019 PMC3370882

[16] Kampfenkel K, Braun V. Membrane topology of the Escherichia coli ExbD protein. Journal of bacteriology. 1992;174(16):5485–7. 1644779 10.1128/jb.174.16.5485-5487.1992 PMC206394

[17] Sverzhinsky A, Fabre L, Cottreau AL, Biot-Pelletier DM, Khalil S, Bostina M, et al Coordinated rearrangements between cytoplasmic and periplasmic domains of the membrane protein complex ExbB-ExbD of Escherichia coli. Structure. 2014;22(5):791–7. 10.1016/j.str.2014.02.010 24657092

[18] Gresock MG, Savenkova MI, Larsen RA, Ollis AA, Postle K. Death of the TonB Shuttle Hypothesis. Front Microbiol. 2011;2:206 10.3389/fmicb.2011.00206 22016747 PMC3191458

[19] Kaserer WA, Jiang X, Xiao Q, Scott DC, Bauler M, Copeland D, et al Insight from TonB hybrid proteins into the mechanism of iron transport through the outer membrane. J Bacteriol. 2008;190(11):4001–16. 10.1128/JB.00135-08 18390658 PMC2395051

[20] Kojima S, Blair DF. Conformational change in the stator of the bacterial flagellar motor. Biochemistry. 2001;40(43):13041–50. 11669642 10.1021/bi011263o

[21] Jordan LD, Zhou Y, Smallwood CR, Lill Y, Ritchie K, Yip WT, et al Energy-dependent motion of TonB in the Gram-negative bacterial inner membrane. Proc Natl Acad Sci U S A. 2013;110(28):11553–8. 10.1073/pnas.1304243110 23798405 PMC3710835

[22] Oddershede L, Dreyer JK, Grego S, Brown S, Berg-Sorensen K. The motion of a single molecule, the lambda-receptor, in the bacterial outer membrane. Biophysical journal. 2002;83(6):3152–61. 10.1016/S0006-3495(02)75318-6 12496085 PMC1302393

[23] Bakshi S, Bratton BP, Weisshaar JC. Subdiffraction-limit study of Kaede diffusion and spatial distribution in live Escherichia coli. Biophys J. 2011;101(10):2535–44. PubMed Central PMCID: PMCPMC3218334. 10.1016/j.bpj.2011.10.013 22098753 PMC3218334

[24] English BP, Hauryliuk V, Sanamrad A, Tankov S, Dekker NH, Elf J. Single-molecule investigations of the stringent response machinery in living bacterial cells. Proc Natl Acad Sci U S A. 2011;108(31):E365–73. PubMed Central PMCID: PMCPMC3150888. 10.1073/pnas.1102255108 21730169 PMC3150888

[25] Lill Y, Kaserer WA, Newton SM, Lill M, Klebba PE, Ritchie K. Single-molecule study of molecular mobility in the cytoplasm of Escherichia coli. Phys Rev E Stat Nonlin Soft Matter Phys. 2012;86(2 Pt 1):021907. 23005785 10.1103/PhysRevE.86.021907

[26] Oh D, Yu Y, Lee H, Wanner BL, Ritchie K. Dynamics of the serine chemoreceptor in the Escherichia coli inner membrane: a high-speed single-molecule tracking study. Biophys J. 2014;106(1):145–53. PubMed Central PMCID: PMCPMC3907255. 10.1016/j.bpj.2013.09.059 24411246 PMC3907255

[27] Leake MC, Chandler JH, Wadhams GH, Bai F, Berry RM, Armitage JP. Stoichiometry and turnover in single, functioning membrane protein complexes. Nature. 2006;443(7109):355–8. 10.1038/nature05135 16971952

[28] Leake MC, Greene NP, Godun RM, Granjon T, Buchanan G, Chen S, et al Variable stoichiometry of the TatA component of the twin-arginine protein transport system observed by in vivo single-molecule imaging. Proc Natl Acad Sci U S A. 2008;105(40):15376–81. PubMed Central PMCID: PMCPMC2563114. 10.1073/pnas.0806338105 18832162 PMC2563114

[29] Rassam P, Copeland NA, Birkholz O, Tóth C, Chavent M, Duncan AL, et al Supramolecular assemblies underpin turnover of outer membrane proteins in bacteria. Nature. 2015;523(7560):333–6. PubMed Central PMCID: PMCPMC4905513. 10.1038/nature14461 26061769 PMC4905513

[30] Haas BL, Matson JS, DiRita VJ, Biteen JS. Single-molecule tracking in live Vibrio cholerae reveals that ToxR recruits the membrane-bound virulence regulator TcpP to the toxT promoter. Mol Microbiol. 2015;96(1):4–13. 10.1111/mmi.12834 25318589 PMC6025817

[31] Higgs PI, Larsen RA, Postle K. Quantification of known components of the Escherichia coli TonB energy transduction system: TonB, ExbB, ExbD and FepA. Mol Microbiol. 2002;44(1):271–81. 11967085 10.1046/j.1365-2958.2002.02880.x

[32] Newton SM, Igo JD, Scott DC, Klebba PE. Effect of loop deletions on the binding and transport of ferric enterobactin by FepA. Mol Microbiol. 1999;32(6):1153–65. 10383757 10.1046/j.1365-2958.1999.01424.x

[33] Scott DC, Cao Z, Qi Z, Bauler M, Igo JD, Newton SM, et al Exchangeability of N termini in the ligand-gated porins of Escherichia coli. J Biol Chem. 2001;276(16):13025–33. 10.1074/jbc.M011282200 11278876

[34] Newton SM, Trinh V, Pi H, Klebba PE. Direct measurements of the outer membrane stage of ferric enterobactin transport: postuptake binding. J Biol Chem. 2010;285(23):17488–97. 10.1074/jbc.M109.100206 20335169 PMC2878513

[35] Ritchie K, Shan XY, Kondo J, Iwasawa K, Fujiwara T, Kusumi A. Detection of non-Brownian diffusion in the cell membrane in single molecule tracking. Biophysical journal. 2005;88(3):2266–77. 10.1529/biophysj.104.054106 15613635 PMC1305276

[36] Hashimoto-Gotoh T, Franklin FC, Nordheim A, Timmis KN. Specific-purpose plasmid cloning vectors. I. Low copy number, temperature-sensitive, mobilization-defective pSC101-derived containment vectors. Gene. 1981;16(1–3):227–35. 6282694 10.1016/0378-1119(81)90079-2

[37] Klebba PE, McIntosh MA, Neilands JB. Kinetics of biosynthesis of iron-regulated membrane proteins in Escherichia coli. J Bacteriol. 1982;149(3):880–8. 6174499 10.1128/jb.149.3.880-888.1982 PMC216474

[38] Smallwood CR, Jordan L, Trinh V, Schuerch DW, Gala A, Hanson M, et al Concerted loop motion triggers induced fit of FepA to ferric enterobactin. J Gen Physiol. 2014;144(1):71–80. 10.1085/jgp.201311159 24981231 PMC4076525

[39] Smallwood CR, Marco AG, Xiao Q, Trinh V, Newton SM, Klebba PE. Fluoresceination of FepA during colicin B killing: effects of temperature, toxin and TonB. Mol Microbiol. 2009;72(5):1171–80. 10.1111/j.1365-2958.2009.06715.x 19432807 PMC3082853

[40] Murphy CK, Kalve VI, Klebba PE. Surface topology of the Escherichia coli K-12 ferric enterobactin receptor. Journal of bacteriology. 1990;172(5):2736–46. 2139651 10.1128/jb.172.5.2736-2746.1990 PMC208919

[41] Wayne R, Frick K, Neilands JB. Siderophore protection against colicins M, B, V, and Ia in Escherichia coli. Journal of bacteriology. 1976;126(1):7–12. 131121 10.1128/jb.126.1.7-12.1976 PMC233253

[42] Neidhardt FC, Bloch PL, Smith DF. Culture medium for enterobacteria. Journal of bacteriology. 1974;119(3):736–47. 4604283 10.1128/jb.119.3.736-747.1974 PMC245675

[43] Gelles J, Schnapp BJ, Sheetz MP. Tracking kinesin-driven movements with nanometre-scale precision. Nature. 1988;331(6155):450–3. 10.1038/331450a0 3123999

[44] Kusumi A, Sako Y, Yamamoto M. Confined lateral diffusion of membrane receptors as studied by single particle tracking (nanovid microscopy). Effects of calcium-induced differentiation in cultured epithelial cells. Biophys J. 1993;65(5):2021–40. 10.1016/S0006-3495(93)81253-0 8298032 PMC1225938

[45] Bradbeer C. The proton motive force drives the outer membrane transport of cobalamin in Escherichia coli. Journal of bacteriology. 1993;175(10):3146–50. 8387997 10.1128/jb.175.10.3146-3150.1993 PMC204637

[46] Cao Z, Warfel P, Newton SM, Klebba PE. Spectroscopic observations of ferric enterobactin transport. J Biol Chem. 2003;278(2):1022–8. 10.1074/jbc.M210360200 12409288

[47] Bean GJ, Flickinger ST, Westler WM, McCully ME, Sept D, Weibel DB, et al A22 disrupts the bacterial actin cytoskeleton by directly binding and inducing a low-affinity state in MreB. Biochemistry. 2009;48(22):4852–7. 10.1021/bi900014d 19382805 PMC3951351

[48] Young KD. Bacterial shape: two-dimensional questions and possibilities. Annu Rev Microbiol. 2010;64:223–40. 10.1146/annurev.micro.112408.134102 20825347 PMC3559087

[49] Deich J, Judd EM, McAdams HH, Moerner WE. Visualization of the movement of single histidine kinase molecules in live Caulobacter cells. Proc Natl Acad Sci U S A. 2004;101(45):15921–6. 10.1073/pnas.0404200101 15522969 PMC528753

[50] Gibbs KA, Isaac DD, Xu J, Hendrix RW, Silhavy TJ, Theriot JA. Complex spatial distribution and dynamics of an abundant Escherichia coli outer membrane protein, LamB. Mol Microbiol. 2004;53(6):1771–83. 10.1111/j.1365-2958.2004.04242.x 15341654

