# Supplementary figures and images for: Confined Mobility of TonB and FepA in Escherichia coli Membranes
Source: PLoS One. 2016 Dec 9;11(12):e0160862. doi: 10.1371/journal.pone.0160862 (PMC5147803; doi:10.1371/journal.pone.0160862)

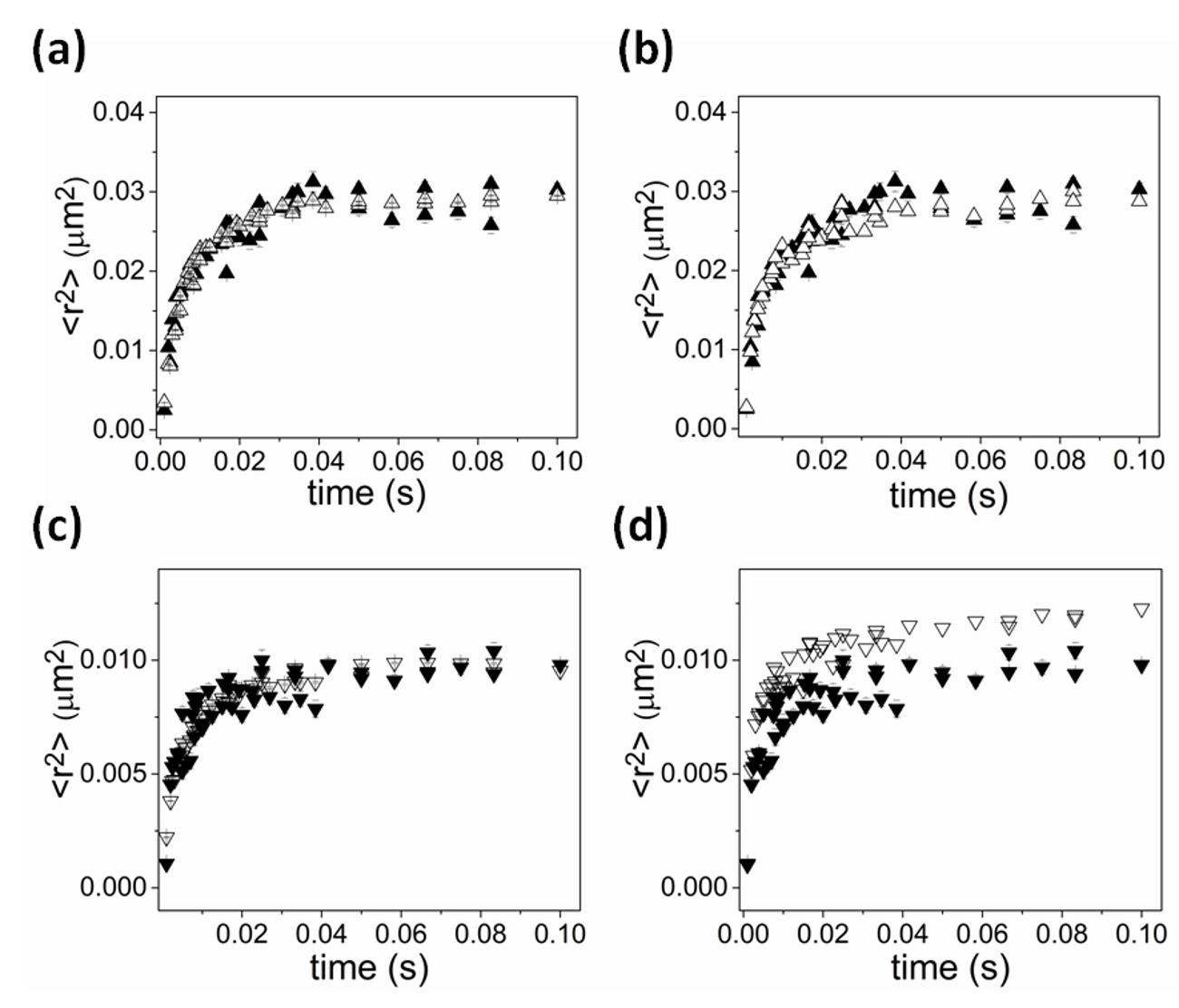

Supplement: S1 Fig — Fit to the MSD of TonB (data: solid triangles, fit: open triangles) by (a) confinement model and (b) assuming a mixture of the MSD for ligand-free TonB (Fig 3B) and FepA (Fig 3C). Fit to the MSD of FepA (data: solid upside-down triangles, fit: open upside-down triangles) (c) confinement model and (d) assuming a mixture of the MSD for ligand-free TonB (Fig 3B) and FepA (Fig 3C). (TIF) [file pone.0160862.s001.tif]

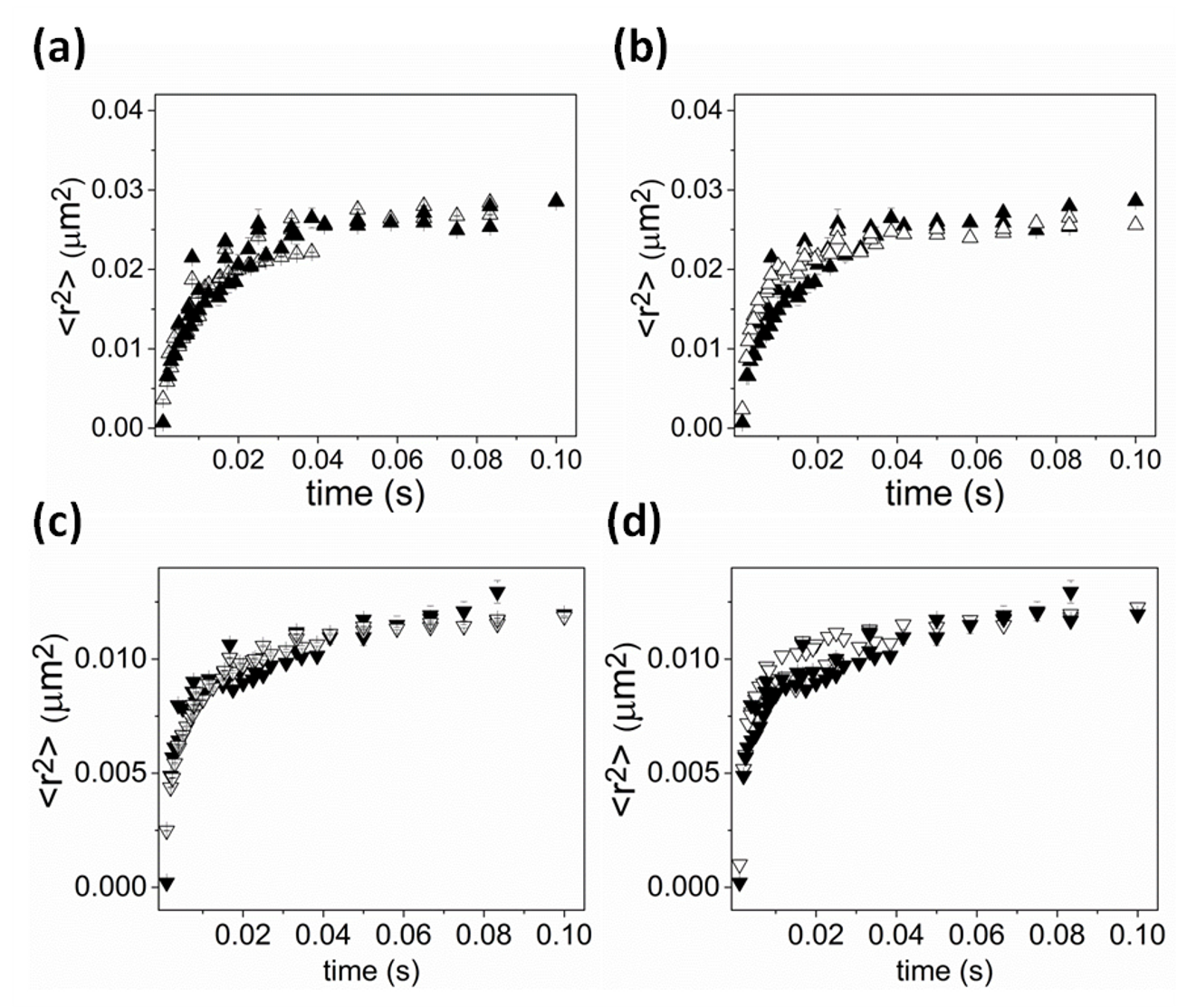

Supplement: S2 Fig — Fit to the MSD of TonB (data: solid triangles, fit: open triangles) by (a) confinement model and (b) assuming a mixture of the MSD for ligand-free TonB (Fig 3B) and FepA (Fig 3C). Fit to the MSD of FepA (data: solid upside-down triangles, fit: open upside-down triangles) (c) confinement model and (d) assuming a mixture of the MSD for ligand-free TonB (Fig 3B) and FepA (Fig 3C). (TIF) [file pone.0160862.s002.tif]

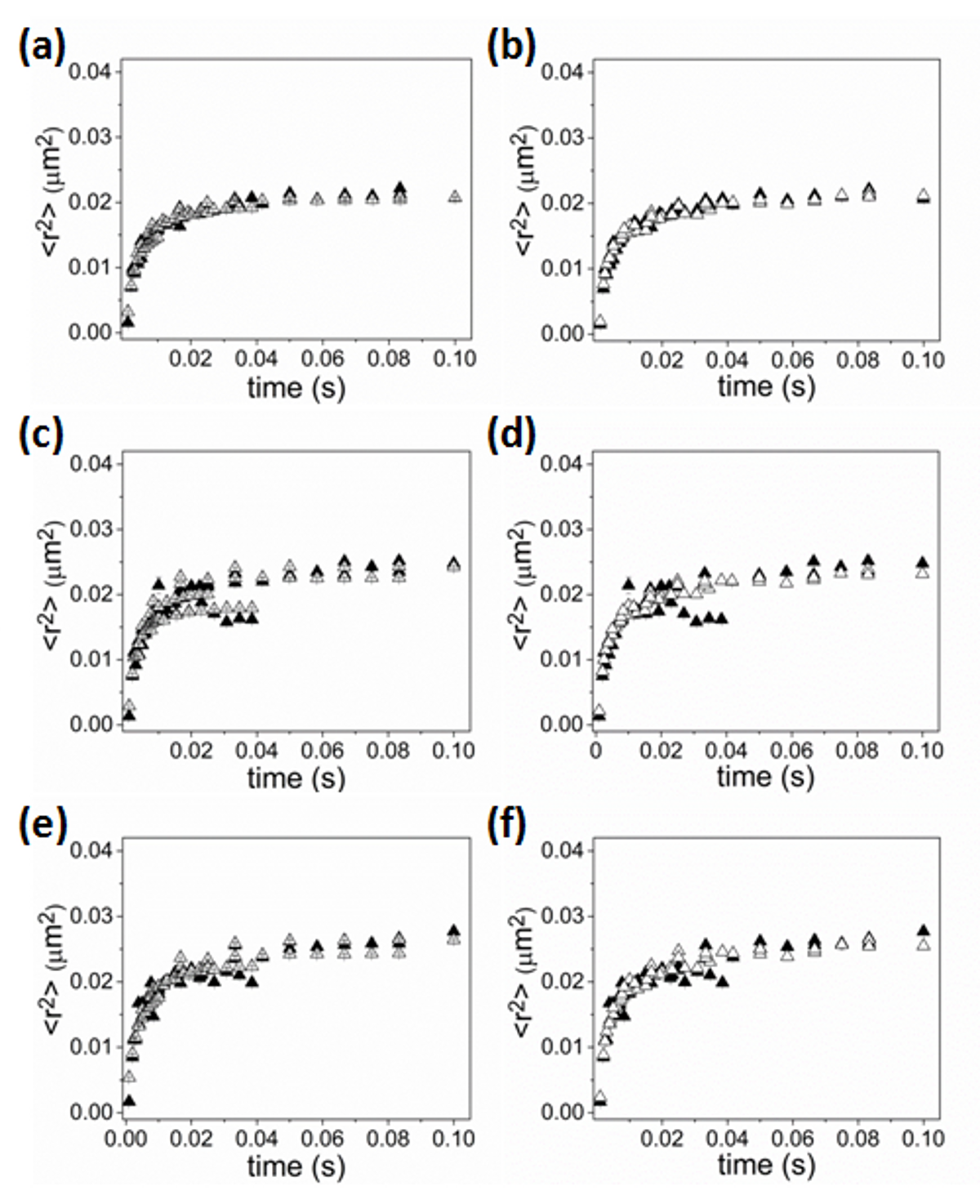

Supplement: S3 Fig — Fit to the MSD of TonB (data: solid triangles, fit: open triangles) by (a,c,e) confinement model and (b,d,e) assuming a mixture of the MSD for ligand-free TonB (Fig 3B) and FepA (Fig 3C) for cells (a,b) lacking ExbB/D, (c,d) in the presence of anti-FepA and (e,f) in the presence of MreB disrupting A22. (TIF) [file pone.0160862.s003.tif]

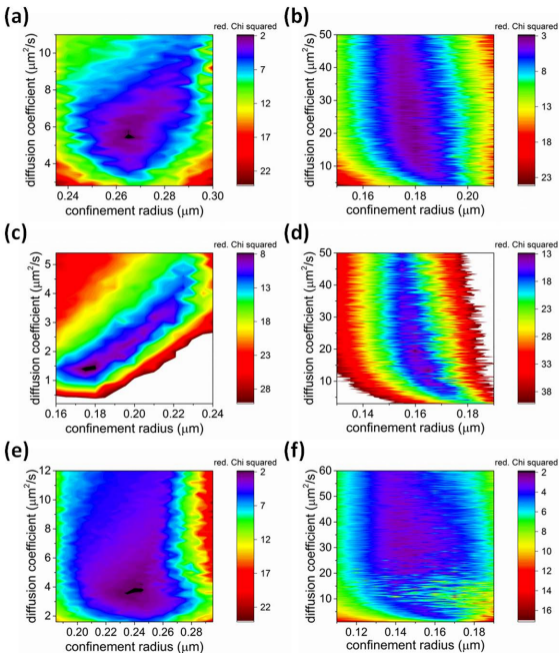

**(g)**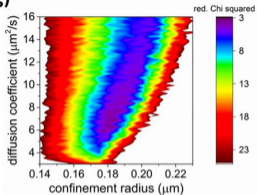**(h)**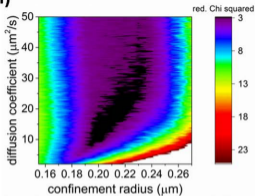**(i)**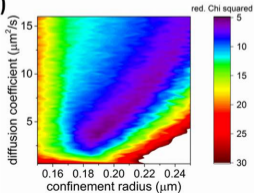

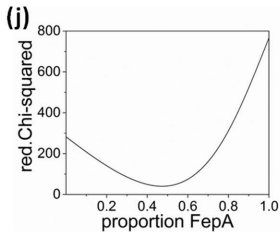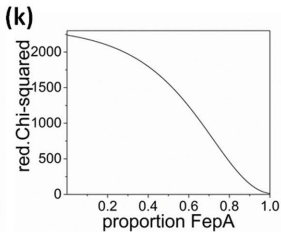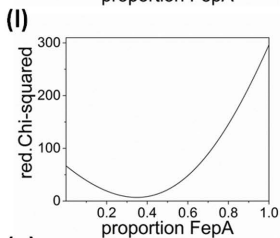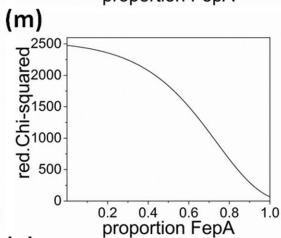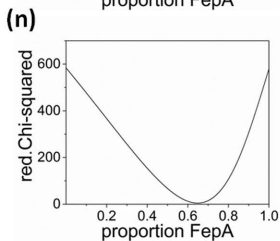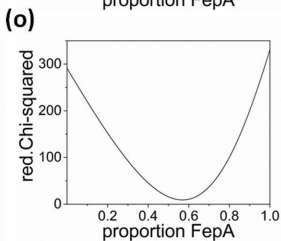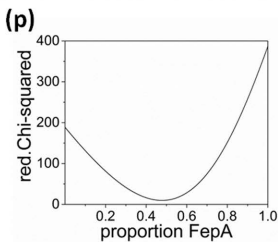

Supplement: S4 Fig — Reduced chi-squared surfaces from Monte Carlo simulation fits to the observed MSDs: ligand-free (a) TonB and (b) FepA, (c, d) TonB and FepA in the presence of FeEnt, (e, f) TonB and FepA in the cells treated with CCCP, (g) TonB for the cells lacking ExbB/D, and TonB in the presence of (h) anti-FepA antibody and (i) A22. Reduced chi-squared distribution for models assuming a mixture of the MSD for ligand-free TonB (Fig 3B) and FepA (Fig 3C): (j, k) TonB and FepA in the presence of FeEnt, (l, m) TonB and FepA in the cells treated with CCCP, (n) TonB for the cells lacking ExbB/D, and TonB in the presence of (o) anti-FepA antibody and (p) A22. (PDF) [file pone.0160862.s004.pdf]
